# The Pathogenesis-Related Maize Seed (*PRms*) Gene Plays a Role in Resistance to *Aspergillus flavus* Infection and Aflatoxin Contamination

**DOI:** 10.3389/fpls.2017.01758

**Published:** 2017-10-17

**Authors:** Rajtilak Majumdar, Kanniah Rajasekaran, Christine Sickler, Matthew Lebar, Bryan M. Musungu, Ahmad M. Fakhoury, Gary A. Payne, Matt Geisler, Carol Carter-Wientjes, Qijian Wei, Deepak Bhatnagar, Jeffrey W. Cary

**Affiliations:** ^1^Food and Feed Safety Research Unit, United States Department of Agriculture – Agricultural Research Service, Southern Regional Research Center, New Orleans, LA, United States; ^2^Department of Plant Biology, Southern Illinois University, Carbondale, IL, United States; ^3^Warm Water Aquaculture Unit, United States Department of Agriculture – Agricultural Research Service, Stoneville, MS, United States; ^4^Department of Plant, Soil and Agriculture Systems, Southern Illinois University, Carbondale, IL, United States; ^5^Department of Plant Pathology, North Carolina State University, Raleigh, NC, United States

**Keywords:** *PRms*, RNAi, *Aspergillus flavus*, maize, aflatoxin resistance, gene network analysis

## Abstract

*Aspergillus flavus* is an opportunistic plant pathogen that colonizes and produces the toxic and carcinogenic secondary metabolites, aflatoxins, in oil-rich crops such as maize (*Zea mays ssp. mays* L.). Pathogenesis-related (PR) proteins serve as an important defense mechanism against invading pathogens by conferring systemic acquired resistance in plants. Among these, production of the PR maize seed protein, *ZmPRms* (AC205274.3_FG001), has been speculated to be involved in resistance to infection by *A. flavus* and other pathogens. To better understand the relative contribution of *ZmPRms* to *A. flavus* resistance and aflatoxin production, a seed-specific RNA interference (RNAi)-based gene silencing approach was used to develop transgenic maize lines expressing hairpin RNAs to target *ZmPRms*. Downregulation of *ZmPRms* in transgenic kernels resulted in a ∼250–350% increase in *A. flavus* infection accompanied by a ∼4.5–7.5-fold higher accumulation of aflatoxins than control plants. Gene co-expression network analysis of RNA-seq data during the *A. flavus*-maize interaction identified *ZmPRms* as a network hub possibly responsible for regulating several downstream candidate genes associated with disease resistance and other biochemical functions. Expression analysis of these candidate genes in the *ZmPRms*–RNAi lines demonstrated downregulation (vs. control) of a majority of these *ZmPRms*-regulated genes during *A. flavus* infection. These results are consistent with a key role of *ZmPRms* in resistance to *A. flavus* infection and aflatoxin accumulation in maize kernels.

## Introduction

Mycotoxin contamination of food and feed crops is a global problem. Exposure to mycotoxins in humans and livestock primarily occurs through ingestion of contaminated seeds or other edible plant parts. The majority of mycotoxin contamination of crop plants is the result of infection by members of the three fungal genera, *Aspergillus*, *Fusarium*, and *Penicillium* among which *Aspergillus* is responsible for the most adverse economic and health impacts (Ismaiel and Papenbrock, 2015; Kumar et al., 2016; Mitchell et al., 2016; Umesha et al., 2017). Maize is a major crop grown worldwide and is susceptible to aflatoxin contamination by *Aspergillus flavus*, especially during episodes of severe drought (Kebede et al., 2012; Fountain et al., 2014). Aflatoxin contamination of maize can result in economic losses as high as US$686.6 million/year in the United States based on a recent estimation of economic losses for the year 2013 (Mitchell et al., 2016). With predicted changes in the global climate, it is estimated that aflatoxin contamination could cause losses to the maize industry ranging from US$52.1 million to US$1.68 billion/year in the United States (Mitchell et al., 2016).

Production of pathogenesis-related (PR) proteins in response to biotic stressors is a defense strategy often employed by plants to resist pathogen invasion. PR proteins inhibit pathogens either by their direct antimicrobial properties or by regulating the expression of key genes involved in host defense or both. This class of defense proteins has been shown to accumulate at the site of infection upon pathogen invasion and contribute to systemic acquired resistance (SAR) (Ryals et al., 1996). Transcriptomic and proteomic studies have identified changes in protein production during maize seed infection by *A. flavus* and *Fusarium verticillioides* (reviewed by Pechanova and Pechan, 2015). Among the different PR proteins reported, PR10 (GenBank accession no. AY953127) has been extensively studied in corn in relation to *A. flavus* infection (Chen et al., 2010). Silencing of PR10 (under constitutive promoter) in maize resulted in a significant increase in fungal growth in the kernels accompanied by higher amounts of aflatoxin accumulation. The contribution of PR10 to aflatoxin resistance in maize has been mainly attributed to the antimicrobial property of the PR10 protein. The other PR protein that is also highly induced upon infection by necrotrophic seed pathogenic fungi is the PR maize seed (*ZmPRms*; AC205274.3_FG001) protein (Casacuberta et al., 1991; Murillo et al., 1999; Dolezal et al., 2014; Shu et al., 2015; Musungu et al., 2016).

*ZmPRms* transcripts are reported to accumulate at the aleurone layer and scutellum of germinating maize seeds (Casacuberta et al., 1991) and their production is increased upon fungal infection (Casacuberta et al., 1992; Murillo et al., 1999; Shu et al., 2015). The involvement of the *ZmPRms* gene promoter in response to fungal elicitors was also reported by Raventós et al. (1995). *ZmPRms* promoter::reporter gene fusion assays demonstrated activation of the promoter by fungal elicitors (mycelial extract derived from *F. verticillioides*). Presence of a specific motif in the promoter region showed strong association between promoter induction and biotic stressors. Involvement of both elicitor-response element (ERE) and enhancing sequences in the *PRms* promoter were implicated in induction of the promoter by fungal elicitors. In another study, transgenic expression of the *ZmPRms* gene in rice resulted in broad-spectrum resistance against fungal (*Magnaporthe oryzae*, *F. verticillioides*, and *Helminthosporium oryzae*) and bacterial (*Erwinia chrysanthemi*) pathogens suggesting involvement of this gene in the central defense mechanism in plants (Gómez-Ariza et al., 2007). Overexpression of *ZmPRms* in rice also primed the expression of other pathogen-induced defense genes leading to significantly higher expression at the basal level (in absence of the pathogen) and higher induction upon pathogen exposure as compared to the wild-type rice plants.

Both marker-assisted breeding and transgenic approaches are being used to develop maize lines with enhanced resistance to *A. flavus* infection and aflatoxin contamination (Cary et al., 2011; Warburton and Williams, 2014; Bhatnagar-Mathur et al., 2015; Schubert et al., 2015; Brown et al., 2016; Thakare et al., 2017). Both of these approaches require identification of host genes that contribute to enhanced resistance. The objective of the current study was to determine and evaluate the relative contribution of *ZmPRms* against *A. flavus* infection and aflatoxin production during infection of maize kernels. Using an RNAi-based approach, transgenic maize plants were generated with seed-specific expression of hairpin RNAs (hpRNA) to silence the *ZmPRms* gene. Downregulation in expression of the native *ZmPRms* gene in selected transgenic RNAi maize lines allowed us to quantify the role of *ZmPRms* in fungal growth and aflatoxin accumulation in infected kernels. We also evaluated *ZmPRms*-mediated global regulation of host defense-related genes and other biochemical function-related genes associated with resistance to *A. flavus* in maize.

## Materials and Methods

### *ZmPRms*–RNAi Vector Construction

**FIGURE 1:**
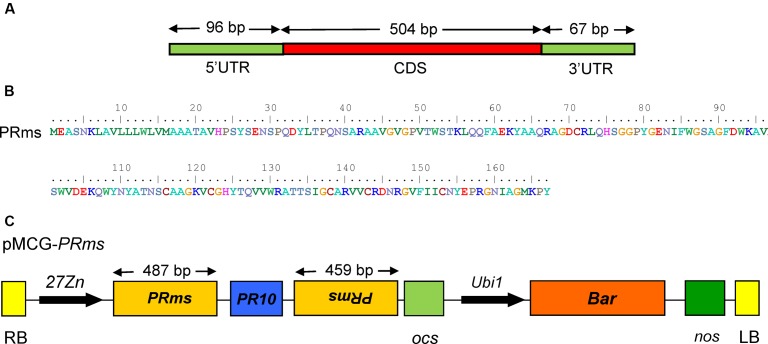
*ZmPRms* gene and vector construction. **(A)** Genomic structure of the *Zea mays* (*Zm*) *PRms* gene; **(B)** Amino acid sequence of the *ZmPRms* gene; **(C)** Vector diagram of the RNAi construct used for maize transformation to silence the *ZmPRms* gene (Abbreviations: 27*Zn* = maize endosperm-specific promoter, RB = right border, LB = left border, PR10 = intron (maize), *Ubi*1 = constitutive promoter, *Bar* = bialaphos resistance gene, *ocs* and *nos* = transcription terminators).

An RNAi-based binary vector for host-induced gene silencing (HIGS) of the *ZmPRms* gene (**Figures 1A,B**) was constructed using an In-fusion HD Cloning Kit (Clontech Laboratories, Inc.; Cat# 011614). Briefly, a 1115 bp *Zein* promoter (Shepherd and Scott, 2009), a 460 bp 5′ arm (the sense strand) and a 454 bp 3′ arm (the antisense strand) of the *ZmPRms* gene and a PR10 intron (Chen et al., 2010) were PCR amplified using sequence-specific primers (Supplementary Table S1) containing a 15 bp overlap at the 5′ ends. The three PCR fragments were joined together with *ScaI-SpeI* restriction enzyme digested pMCG1005 binary plant transformation vector (McGinnis et al., 2005) using the In-Fusion^®^ HD Cloning Kit to generate the vector, pMCG–*ZmPRms*–RNAi (**Figure 1C**). The PR10 intron inserted between the sense and antisense *ZmPRms* fragments facilitated efficient splicing and formation of hpRNA in the transgenic *ZmPRms*–RNAi plants.

### Maize Transformation and Plant Growth Conditions

*Agrobacterium*-mediated transformation of maize (inbred B104) with the pMCG–*ZmPRms*–RNAi vector was carried out at the Iowa State University Plant Transformation Center (Frame et al., 2011). Several independent transgenic events were regenerated for further analysis. Putative transgenic plants and their progenies were grown in moist soil mix containing three parts Scott’s 360 Metro-Mix (Scotts Company, Marysville, OH, United States) and one part perlite in 3^′′^ (7.6 cm) pots. Seedlings were first grown in a growth chamber at 25°C under 16-h photoperiod (80 μmol m^-2^ s^-1^) for 4 weeks prior to transfer to 5 gal (0.02 cubic meter) pots in the greenhouse (27 ± 2°C).

### PCR Screening

Plants were screened by PCR using the ‘Phire Plant Direct PCR Kit’ (ThermoFisher Scientific; Cat# F160S) according to the manufacturer’s protocol. Primers used to screen putative*ZmPRms*–RNAi plants for the presence of the RNAi transgene cassette were 27zn945-F 5′-ccatgaagctgcctacagc-3′ and PR10_R 5′-cggaattccgtatggcaa-3′, while primers *Bar*_F: 5′-caccatcgtcaaccactacatcgagac-3′ and *Bar*_R: 5′-cagctgccagaaacccacgtcatgc-3′ (Rajasekaran et al., 2017) were used to screen empty vector transformed control plants. A 55°C annealing temperature and 30 s elongation time were used to amplify 1156 bp and 433 bp fragments that confirmed the presence of the RNAi expression cassette and the *bar* gene, respectively, in the transgenic plants.

### Fungal Strain and Inoculum Preparation

An aflatoxin-producing *A. flavus* 70 strain expressing GFP (AF70-GFP; Rajasekaran et al., 2008) was obtained from the SRRC fungal collection (SRRC 1436; ARS-USDA, New Orleans, LA, United States). The fungal strain was grown on 2x concentrated V8 agar media (2x V8; Cary et al., 2015) for 7 days at 30°C with illumination. Conidia were harvested by flooding each plate with 20 ml of 0.02% (v/v) sterile Triton X-100 solution and gently dislodging conidia from the surface mycelia with a sterile scraper. Conidial suspensions were adjusted to 4 × 10^6^ spores/ml prior to the kernel inoculation.

### Kernel Inoculation and Incubation

Undamaged and roughly uniform size T_1_ maize kernels (B104) collected from transgenic *ZmPRms*–RNAi and control plant lines were randomly assigned and processed according to a kernel-screening assay (KSA; Rajasekaran et al., 2013). All kernels were surface sterilized in 70% ethanol, air dried, and stored in sterile tubes. Kernels were inoculated by immersion into a 4 × 10^6^ spores/ml suspension of the AF70-GFP strain followed by stirring for 3 min. The inoculum was then drained off and the kernels were transferred to plastic caps that were placed in a tray for each transgenic line. The kernels were incubated under high RH ( > 90%) at 31°C for 7 days in the dark. The filter paper inside the tray was kept moist by adding extra water when needed during the incubation period.

### Digital Imaging and Quantification of GFP Fluorescence

AF70-GFP infected maize kernels were harvested 7 days post inoculation (dpi), longitudinally sectioned, and photographed using a stereomicroscope (Nikon SMZ25, Melville, NY, United States) equipped with a GFP filter and a camera to capture images of GFP fluorescence (excitation 485 nm, emission 528 nm). Individual kernels frozen in liquid nitrogen were homogenized using a SPEX SamplePrep (Geno/Grinder, Metuchen, NJ, United States) and the ground seed samples (∼25 mg fresh weight) were extracted in 500 μl of Sorenson’s phosphate buffer (pH 7.0). Samples were vortexed for 30 s followed by centrifugation at 9000 × *g* for 15 min. A 100 μl aliquot of the supernatant was carefully transferred to a 96-well plate and GFP fluorescence was measured at an excitation wavelength of 485 nm and an emission wavelength of 535 nm using a fluorometer (BioTek, Synergy4, Winooski, VT, United States) along with the appropriate control (buffer only blank).

### Aflatoxin Analysis

Homogenized maize kernel tissue (∼20–70 mg) was extracted with ethyl acetate/acetone (1:1)/0.1 % formic acid (1 ml) for 24 h at room temperature. The extracts were filtered through cotton plugs and the filtrates were concentrated under nitrogen to dryness. Each extract was re-dissolved in acetonitrile (1 mg/ml), filtered through a 0.22 μm Spin-X centrifuge tube filter, and analyzed on a Waters Acquity UPLC system (40% MeOH in water, BEH C18 1.7μm, 2.1 × 50 mm column) using fluorescence detection (Ex = 365 nm, Em = 440 nm). Samples were diluted 10-fold if the aflatoxin signal saturated the detector. Analytical standards (Sigma–Aldrich, St. Louis, MO, United States) were used to identify and quantify aflatoxins: aflatoxin B1 (AFB1, retention time = 4.60 min.); aflatoxin B2 (AFB2, retention time = 3.55 min.). Aflatoxin contents were expressed in ng/mg fresh weight of homogenized maize kernels.

### RNA-Seq Data Mining and Gene Regulatory Network Analysis

The data used to identify potential maize genes involved in resistance to *A. flavus* infection and aflatoxin contamination was obtained from a set of RNA-seq data of the *A. flavus*–maize interaction publically available and located at NCBI [SRP082421] (Musungu et al., 2016). Additionally, data from the PiZeam interactome (Musungu et al., 2015) was utilized to detect additional gene targets by analyzing the overlap between the networks. For mining of the RNA-seq data, a two-pronged approach was utilized with PiZeaM, a *Z. mays*–*A. flavus* interactome (Musungu et al., 2015), and a published *A. flavus*/*Z. mays* gene co-expression network (Musungu et al., 2016). First, the RNA-seq data was analyzed as described in Musungu et al. (2016) and large regulatory networks inferred. The ‘R’ statistical language was then used to mine the networks for *ZmPRms*. After filtering by correlation strength and overlap, *ZmPRms* was predicted to be regulating multiple *Z. mays* and *A. flavus* genes. The sub-network was further analyzed using the PiZeaM interactome (Musungu et al., 2015) to identify protein–protein interactions in *Z. mays*, potentially revealing downstream targets of *ZmPRms*. This approach resulted in the identification of additional targets that would have been missed if differential expression had been used as the only metric cutoff to identify partners of *ZmPRms*.

### RNA Isolation, cDNA Synthesis, and Gene Expression Analysis

RNA was isolated from individual homogenized maize kernels using the ‘Spectrum^TM^ Plant Total RNA kit’ (Sigma–Aldrich, St. Louis, MO, United States) and cDNA was synthesized using iScript^TM^ cDNA synthesis kit (Bio-Rad). Quantitative RT-PCR (qRT-PCR) was performed using SYBR green I chemistry and iCycler iQ5 Multicolor real-time PCR detection system (Bio-Rad). The thermocycling conditions included a pre-incubation at 95°C for 3 min, dye activation at 95°C for 10 s, primer annealing at 55°C for 30 s, elongation at 55°C for 50 s followed by a dissociation curve between 65°C and 95°C for 30 min (with 0.5°C increments). The primers used for qRT-PCR are presented in Supplementary Table S1. Gene expression was normalized by ΔΔC_T_ analysis (Livak and Schmittgen, 2001) to *Zea mays* ribosomal structural gene GRMZM2G024838 expression (Shu et al., 2015) utilizing the gene expression analysis software package of the Bio-Rad iQ5.

### Analysis of the *ZmPRms* Promoter

A 700 bp upstream promoter region of the maize *PRms* gene was analyzed using the ‘PlantPAN 2.0’ web tool (Chow et al., 2016) to identify putative transcription factor–binding site motifs present in the promoter region.

### Statistical Analysis

For all experiments, typically three to five biological replicates were used per treatment. The data presented here are mean ± SE. Statistical significance between control and RNAi lines were determined by Student’s *t*-test. Significant difference between treatment and control was analyzed at ^∗∗^*P* ≤ 0.05 and/or ^∗^*P* ≤ 0.1 as indicated in the Figure legends.

## Results

### PCR and qRT-PCR Screening of *ZmPRms*–RNAi and Control Plants

**FIGURE 2:**
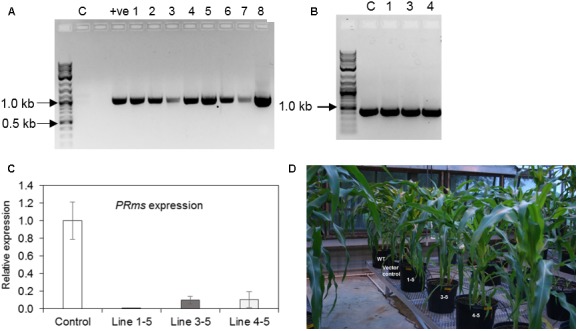
Confirmation of maize transgenic plants and plant phenotype. **(A)** Genomic DNA isolated from *ZmPRms–*RNAi lines and control plant were used to amplify a 1156 bp DNA fragment to confirm the presence of the RNAi cassette (C = empty vector transformed control plants; +ve = plasmid vector used as a PCR template; NEB 2-Log DNA ladder user as a DNA marker); **(B)** Genomic DNA isolated from *ZmPRms–*RNAi and empty vector transformed control (C) plants were used to amplify a 433 bp diagnostic DNA fragment to confirm the presence of the ‘*Bar*’ plant selection marker gene (Line 1 = 1–5, Line 3 = 3–5, Line 4 = 4–5; NEB 2-Log DNA ladder user as a DNA marker); **(C)** Relative expression of the native *PRms* gene in the kernels of empty vector transformed control and *PRms*-RNAi maize lines at 7 days post inoculation (dpi) [Gene expression was normalized to the maize ribosomal structural gene GRMZM2G024838 (Shu et al., 2015); data are mean ± SE of 3–4 biological replicates]; **(D)** Plant growth phenotype (uninfected) of *ZmPRms–*RNAi lines as compared to the wild type (WT) or empty vector transformed control transgenic maize plants.

PCR screening of genomic DNA isolated from the leaves of seven independent transgenic events of *ZmPRms*–RNAi plants were initially confirmed for the presence of an 1156 bp product spanning the 27 *Zein* promoters to the PR10 intron region of the *ZmPRms*–RNAi cassette (**Figure 2A**). Genomic DNA from both *ZmPRms*–RNAi and empty vector control plants amplified a region of 433 bp specific to the plant selection marker gene ‘*Bar*’ (**Figure 2B**). Based on the screening of fungal growth in the *ZmPRms*–RNAi silenced lines, three independent *ZmPRms*–RNAi lines that showed higher fungal growth were selected for further investigation to understand the impact of seed-specific *ZmPRms* silencing on *A. flavus* growth and aflatoxin production. Expression of the native *ZmPRms* gene in transgene positive individual seeds of RNAi lines and empty vector control plants inoculated with the AF70-GFP strain were examined by qRT-PCR. Seed-specific RNAi-mediated silencing of the native *ZmPRms* gene resulted in a ∼90–99% downregulation in expression of the gene (**Figure 2C**) in seeds of infected *ZmPRms*–RNAi lines compared to seeds of infected control lines. Line 1-5 showed the lowest relative expression (0.001) followed by the line 4–5 (0.104) and line 3–5 (0.100). Transgenic *ZmPRms*–RNAi plants were comparable to the wild type (WT) or empty vector transformed control plants with respect to plant morphology (**Figure 2D**).

### Analysis of *Aspergillus flavus* Growth

**FIGURE 3:**
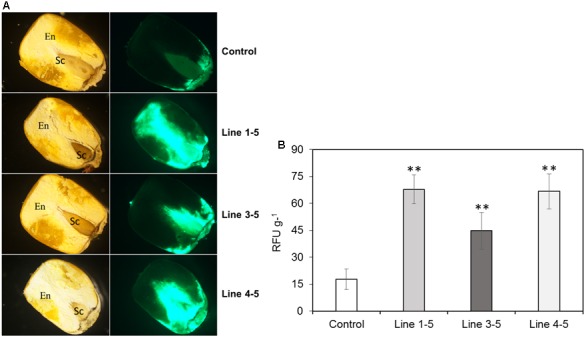
Fungal growth in the T1 generation maize kernels. **(A)** Growth of *A. flavus*-GFP at 7 dpi (as indicated by relative GFP fluorescence; Rajasekaran et al., 2013) in empty vector transformed control and *ZmPRms–*RNAi transgenic maize kernels (First column: light micrographs and second column: GFP fluorescence micrographs of longitudinal sections of kernels. En: endosperm; Sc: scutellum); **(B)** Quantification of GFP fluorescence in the kernels of empty vector transformed control and *ZmPRms–*RNAi maize transgenic plants at 7 days post inoculation (dpi) with *A. flavus*. Relative fluorescence units (RFU) is directly proportional to fungal growth in the kernels. Data are mean ± SE of 5 biological replicates. ^∗∗^Denotes significant difference between control and *PRms* silenced lines (*P* ≤ 0.05).

Fungal growth in the seeds from *ZmPRms*–RNAi plant lines compared to control plants was qualitatively analyzed by fluorescence microscopy of longitudinally sectioned seeds (**Figure 3A**). Imaging of seed from the three *ZmPRms*–RNAi lines showed a significantly higher degree of GFP fluorescence and spread compared to seed from an empty vector control. Visual evidence of increased GFP fluorescence, primarily in the scutellar and adjacent endosperm tissue of the seed-correlated well with the data obtained from absolute quantification of GFP fluorescence. An increase of ∼250–350% in GFP fluorescence was observed in the *ZmPRms*–RNAi lines compared to the control with lines 1–5 showing the greatest increase followed by lines 4–5 and 3–5 (**Figure 3B**).

### Analysis of Aflatoxin Production

**FIGURE 4:**
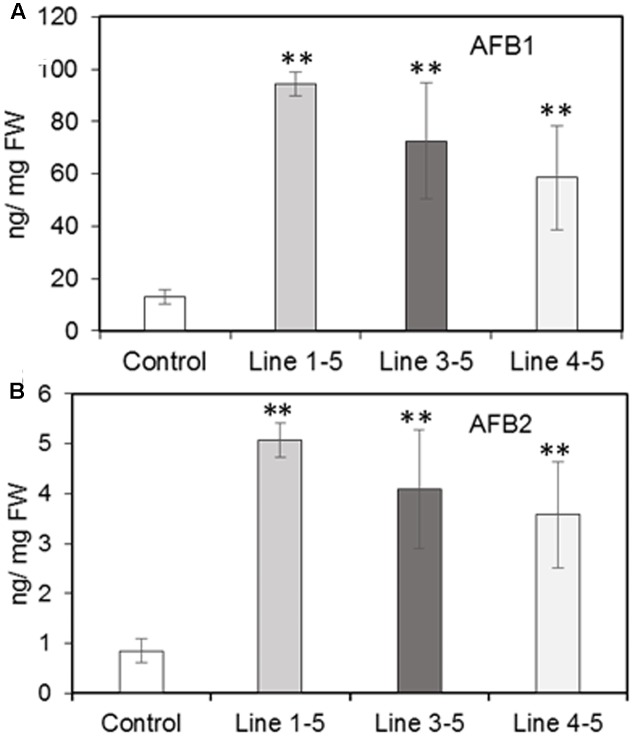
Aflatoxin (AF) contents in the control (empty vector transformed) and *ZmPRms–*RNAi transgenic maize kernels at 7 days post inoculation (dpi) with *A. flavus*. **(A)** AFB1; and **(B)** AFB2. Data are mean ± SE of 4 biological replicates. ^∗∗^Denotes significant difference between control and *PRms* silenced lines (*P* ≤ 0.05).

Silencing of the *PRms* gene in maize significantly affected aflatoxin content in the kernels of silenced lines as compared to the control. In general, the quantities of aflatoxin B1 were several folds higher than those of B2 in both control and silenced lines. A 4.6–7.4-fold increase (significant) in aflatoxin B1 content (ranging between 58 and 94 ng/mg FW) was observed in the *ZmPRms*–RNAi kernels (vs. control; **Figure 4A**) and a 4.2–6.0-fold increase (significant) in aflatoxin B2 content (ranging between 3.5 and 5 ng/mg FW) were observed in the *ZmPRms*–RNAi kernels (vs. control; **Figure 4B**).

### Gene Expression Analysis of *ZmPRms*-Regulated Downstream Target Genes

**FIGURE 5:**
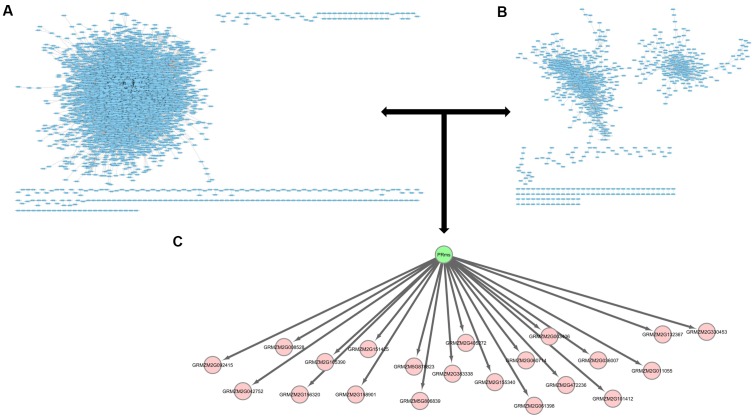
Potential downstream targets of *ZmPRms*. **(A)** The predicted maize interactome (Musungu et al., 2016); **(B)** a maize co-expression network (Musungu et al., 2016); **(C)** potential *ZmPRms* transcriptional targets.

Analysis of the RNA-seq data resulting from the *A. flavus*–maize pathogenic interaction has previously identified *ZmPRms* as a kernel PR protein possibly involved in resistance against *A. flavus* (Musungu et al., 2016). Further gene regulatory network analysis (in the current study) of the previously published RNA-seq data identified downstream gene targets possibly regulated by *ZmPRms* during the *A. flavus*–maize interaction (**Figure 5**, **Table 1** and **Supplementary Figures S1**–**S3**). These included candidates associated with pathogen responses in plants, LRR and NB-ARC domain-containing gene (GRMZM2G060714), RabGAP/TBC-domain containing gene (GRMZM2G156320), Rab28 defense-related gene (GRMZM2G472236), and F-Box gene (GRMZM2G008528). Several other genes belonging to diverse biochemical functions, namely anthocyanidin 3-O-glucosyltransferase (GRMZM2G165390), adenine nucleotide alpha hydrolases-like gene (GRMZM2G151425), inositol monophosphatase (GRMZM2G036007), plasma-membrane choline transporter (GRMZM2G330453), choline transporter (GRMZM2G330453), tRNA-His guanylyltransferase (GRMZM2G158901), and an ATP-binding microtubule motor family gene (GRMZM5G878823), were also identified as *ZmPRms*-regulated genes. Other transcription factor-related candidates included Myb (GRMZM2G003406) and ereb44 (GRMZM5G806839). Several other candidates referred to ‘uncharacterized’ (unknown biological function; **Table 1**) also appeared to be regulated by *ZmPRms*.

**Table 1:** Predicted downstream targets of the *ZmPRms* gene in maize.

Gene ID	Annotation
GRMZM2G165390	Anthocyanidin 3-O-glucosyltransferase
GRMZM2G003406	Putative MYB DNA-binding domain superfamily protein
GRMZM2G008528	F-box containing gene
GRMZM2G011055	Uncharacterized
GRMZM2G036007	Inositol monophosphatase family protein
GRMZM2G042752	Uncharacterized
GRMZM2G060714	LRR and NB-ARC domains-containing disease resistance protein
GRMZM2G061398	Uncharacterized protein
GRMZM2G092415	Uncharacterized protein
GRMZM2G101412	Uncharacterized protein
GRMZM2G132367	HB-type transcription factor
GRMZM2G151425	Adenine nucleotide alpha hydrolases-like superfamily protein
GRMZM2G155340	Uncharacterized protein
GRMZM2G156320	RabGAP/TBC domain-containing protein
GRMZM2G158901	tRNA-His guanylyltransferase
GRMZM2G330453	Plasma-membrane choline transporter family protein
GRMZM2G383338	Uncharacterized
GRMZM2G405272	Uncharacterized
GRMZM5G806839	ereb44 Ap2-erebp-transcription factor 44
GRMZM5G878823	ATP-binding microtubule motor family protein
GRMZM2G472236	Rab28 protein

*ZmPRms was used to mine the gene regulatory network for downstream targets that are likely to be effected. The ‘Gene ID’ is the ‘maizegdb’ ID for the gene of interest and the ‘Annotation’ is the predicted maize annotation or the best Arabidopsis hit*.

**FIGURE 6:**
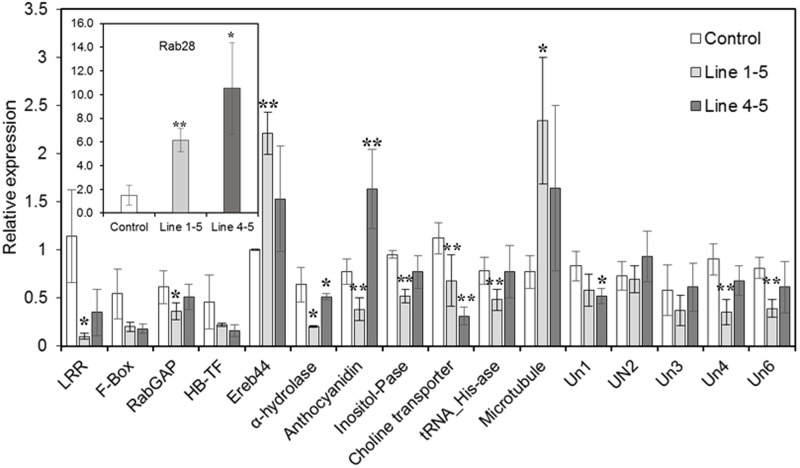
Relative expression of predicted *ZmPRms*-regulated downstream target (obtained from gene regulatory network analysis; **Figure 5** and **Table 1**) candidate genes in the control (empty vector transformed) and *ZmPRms–*RNAi transgenic maize kernels at 7 days post inoculation (dpi) with *A. flavus*. Gene expression was normalized to the maize ribosomal structural gene GRMZM2G024838 (Shu et al., 2015). Data are mean ± SE of 3–4 biological replicates. ^∗∗^/^∗^Denote significant difference between control and *PRms* silenced lines (^∗∗^*P* ≤ 0.05, ^∗^*P* ≤ 0.1). Un1 (Uncharacterized 1; GRMZM2G042752), Un2 (Uncharacterized 2; GRMZM2G061398), Un3 (Uncharacterized 3; GRMZM2G092415), Un4 (Uncharacterized 4; GRMZM2G101412), Un6 (Uncharacterized 6; GRMZM2G383338).

Real-time expression analyses were performed on several of the aforementioned maize genes identified from the gene network analysis, that are possibly regulated by *ZmPRms* during the *A. flavus*–maize interaction (**Table 1**). Several disease resistance/biotic stress response–related genes, namely the LRR and NB-ARC domain-containing gene, RabGAP/TBC-domain containing gene, and F-Box gene, were downregulated in the *ZmPRms*–RNAi lines (**Figure 6**). Other genes associated with diverse biochemical functions including an adenine nucleotide alpha hydrolase-like gene, inositol monophosphatase, plasma-membrane choline transporter, and a tRNA-His guanylyltransferase were also downregulated in the *ZmPRms* silenced lines. Genes that were upregulated in the *ZmPRms*–RNAi lines included ereb44 (transcription factor), an ATP-binding microtubule motor family gene, and a Rab28 defense-related gene among which the Rab28 gene showed the highest upregulation (∼6–10-fold) in the *ZmPRms* silenced lines.

### *In Silico* Analysis of the *ZmPRms* Promoter

Based on the physiological responses of the *PRms* gene in maize, we analyzed the upstream promoter region to identify any stress-related motifs that might help us better understand the biological function of this gene. Analysis of the 700 bp upstream promoter region of the *ZmPRms* gene revealed the presence of several transcription factor–binding motif sites associated with both biotic and abiotic stress responses in plants. These include MYBPLANT, MybSANT, WRKY, ANAERO1CONSENSUS motifs, and several others. Details of the locations, consensus sequences, and physiological functions of these *cis* elements are presented in **Table 2**.

**Table 2:** Putative transcription factor–binding site motifs present in the 700 bp upstream promoter region of the maize *PRms* gene^1^.

Motif name	Consensus sequence	Upstream location	Physiological responses
ANAERO1CONSENSUS	AAACAag/taTGTTT	553, 616	Anaerobic condition
ANAER02CONSENSUS	AGCAGc	13	Anaerobic condition
ANAER03CONSENSUS	TCATCtc	523	Anaerobic condition
ARECOREZMGAPC4	AGCAAtagac	449	Anaerobic condition
DOFCOREZM	AAAGG/AAAGT/AAAGC	195, 255, 456, 562, 681	Transcription regulation
DRE1COREZMRAB17	TCCGAga	79	Drought, ABA response
EIN3	ctaTGCATgt/aaATGCAcct	240, 274, 370, 436	Ethylene signaling
GATA	tCGATCcata	539	Transcription regulation
Homeo domain	ctaTTAATag	115	Transcription regulation
MYBPLANT	cACCTAac, aACCAAac	269, 557, 620	Secondary metabolism
Myb/SANT	tctTATCCg	363	Wounding, drought, salt, cold
SBP	tgatcTGTACaatata	291	Flowering
WRKY	aaTTGACca	291, 314, 354, 363, 464	Pathogen defense, development, secondary metabolism
bZIP	TCATTccttatagtta	518	Transcription regulation (DNA-binding)
Dof	tacTAAAGctg	460	Transcription, translation regulation
TALE KN-1	GGTCA	312, 352	Transcription regulation

^1^*PlantPAN 2.0 plant promoter analysis; Chow et al. (2016)*.

## Discussion

Different approaches have been employed to develop resistance to *A. flavus* infection and aflatoxin contamination in maize. These include conventional breeding approaches that introgress resistance genes into agronomically important maize varieties. Natural sources of genetic resistance against *A. flavus* have been reported in maize but introgression of such resistance into elite germplasm is often hampered with long delays due to the quantitative nature of the trait as well as high phenotypic variability due to genotype–environment interactions (Zila et al., 2013; Warburton and Williams, 2014; Hawkins et al., 2015). As a result, there is a need to evaluate alternative approaches to provide durable resistance. Besides conventional breeding, modern genetic engineering tools have been employed to incorporate *A. flavus* resistance in susceptible maize varieties. These include transgenic expression of natural and synthetic resistance genes and host-induced RNAi-based gene silencing of *A. flavus* genes that are critical in fungal pathogenesis and aflatoxin production (reviewed by Bhatnagar-Mathur et al., 2015; Majumdar et al., 2017; Thakare et al., 2017).

Pathogenesis-related (PR) proteins are products of defense genes, which accumulate at the pathogen infection site and contribute to SAR (reviewed by Fu and Dong, 2013). Among different PR proteins reported in maize kernels, *ZmPRms* is believed to be involved in resistance to fungal pathogens. Murillo et al. (1999), using cellular and subcellular immunolocalization tools, demonstrated the accumulation of the *ZmPRms* protein in the aleurone and inner parenchyma cells of the scutellum post *F. verticillioides* infection. Early activation of the *ZmPRms* protein prior to onset of visual symptoms of *A. flavus* colonization clearly indicated a critical role of *ZmPRms* in kernel resistance to *A. flavus* (Shu et al., 2015). The work described in the present study functionally elucidates the contribution of *ZmPRms* toward *A. flavus* resistance and aflatoxin production during kernel infection. Higher levels of *A. flavus* growth (as determined by GFP fluorescence; **Figure 3A**) was primarily observed in the scutellar and adjacent endosperm tissue and correlated with the downregulation of *ZmPRms* production in *PRms*-RNAi kernels. This supports earlier observations on the spatial distribution of the *ZmPRms* protein in maize seeds during fungal infection (Murillo et al., 1999; Shu et al., 2015). The observed increase in aflatoxin content in the transgenic *ZmPRms*–RNAi maize kernels (**Figure 6**) correlated with increased fungal load in the kernels compared to a control line (**Figure 3**). Earlier work by Raventós et al. (1995) had demonstrated the role of ERE and enhancing element in the *ZmPRms* promoter in response to fungal (*Fusarium sp.*) elicitors. Presence of these *cis* elements in the *ZmPRms* promoter significantly induced the expression of the reporter gene (*Tn9* chloramphenicol acetyltransferase; CAT) when exposed to fungal elicitors. Further *in silico* analysis of the *ZmPRms* promoter in the current study revealed the presence of several stress-related motifs (**Table 2**) associated with biotic/abiotic stress responses in plants (Chow et al., 2016). The role of these putative response elements in *ZmPRms* expression will be the subject of future studies. Besides *ZmPRms*, several other kernel-specific PR proteins are induced upon *A. flavus* infection but only a few are functionally characterized as to their contribution toward aflatoxin resistance (reviewed by Chen et al., 2015; Pechanova and Pechan, 2015). In future studies, it will be interesting to see whether the production of any of these PR proteins is affected due to the downregulation of *ZmPRms* in the kernels of *ZmPRms*–RNAi lines.

Defense response against fungal pathogens in maize is regulated by complex metabolic networks (Musungu et al., 2016). This involves typical transcriptional induction of genes associated with phytohormone-related defense signaling pathways associated with salicylic acid, ethylene, and jasmonic acid (JA) biosynthesis. Both *A. flavus* and *F. verticillioides* were shown to induce these phytohormone-related genes leading to the induction of PR proteins including *ZmPRms* (Murillo et al., 1999; Shu et al., 2015; Tang et al., 2015; Wang et al., 2016). Genome-wide association studies using aflatoxin-resistant inbred maize lines showed a strong correlation between high expression of genes associated with the JA biosynthetic pathway and reduced aflatoxin accumulation in the grains (Tang et al., 2015). Besides JA pathway genes, high expression of other genes, including a leucine-rich repeat protein kinase, expansin B3, reversion-to-ethylene sensitivity1, and an adaptor protein complex2 gene were also reported in the *A. flavus* resistant maize lines. Transcriptomic analysis has identified several candidate genes that are involved in peanut response to *A. flavus* (Wang et al., 2016). The involvement of specific metabolic pathway-related genes in aflatoxin resistance in certain crops/genotypes could be a genotype-related trait. Apparently, *ZmPRms* interacts with different categories of genes to orchestrate resistance against *A. flavus*. Several candidate genes (**Table 1**) identified from gene regulatory network analysis possibly regulated by *ZmPRms* include, genes associated with carbohydrate metabolism (anthocyanidin 3-O-glucosyltransferase), disease resistance (LRR-NB-ARC, RabGAP/TBC, Rab28), transcription factors [Myb, F-Box, and ereb44 (AP2-type)], and other metabolite-related (choline, inositol). Many of these putative *ZmPRms*-regulated genes are known to be induced by *A. flavus* and *F. verticillioides* in maize (Walley et al., 2013; Lanubile et al., 2014; Shu, 2014; Musungu et al., 2016). The majority of these genes were downregulated in the *ZmPRms*–RNAi lines except for a few that were upregulated in the *ZmPRms* silenced lines (**Figure 5**). The upregulated genes include mainly Rab28, ereb44, and microtubule (ATP-binding microtubule motor family protein). The significantly higher expression (∼6–10-fold) of the Rab28 gene in the *ZmPRms*–RNAi lines might indicate a compensatory role in *A. flavus* resistance due to reduced expression of *ZmPRms*. The involvement of many of these candidate genes in disease resistance and abiotic stress tolerance has been reported. Functional characterization (through overexpression in plants) of some of these genes such as Myb, F-Box, LRR, Rab28, and AP2 (ethylene responsive) has resulted in increased resistance against a wide variety of pathogens that include fungi, bacteria, and viruses besides contributing to abiotic stress tolerance in some cases (Cao et al., 2008; Yan et al., 2011; Amara et al., 2013; Jisha et al., 2015; Liu et al., 2016; Xing et al., submitted). Diverse sources of genetic background with substantial aflatoxin resistance have been described in maize (reviewed by Warburton and Williams, 2014; Brown et al., 2016), although, the exact mechanism of such resistance is not fully understood. It will be interesting to see whether any of these *A. flavus* resistant maize genotypes have a positive correlation between higher basal expression and/or rapid activation of *ZmPRms* (in response to *A. flavus*) and increased aflatoxin resistance.

## Conclusion

In this study, we have demonstrated a significant role for *ZmPRms* in resistance to *A. flavus* infection of maize kernels through global regulation of genes associated with biotic and abiotic stress responses in plants. This includes genes associated with disease resistance, carbohydrate metabolism, and transcription factors that are known to be upregulated in plants under stress conditions. The observed increase in *A. flavus* growth and aflatoxin production in the *ZmPRms*–RNAi lines supports the RNA-seq interactome analysis that indicated *ZmPRms* might serve as a major network hub for regulation of downstream resistance-associated gene expression. *ZmPRms*–RNAi lines and their progenies were morphologically normal suggesting that it will be a good candidate host-resistance gene for overexpression in maize for increased resistance to *A. flavus* and possibly against other pathogens. The results presented here are promising and it might be possible to fine tune *ZmPRms* expression in a tissue-specific manner (using modern functional genomic tools) in future or use *ZmPRms* expression as a marker to screen for *A. flavus* resistant maize genotypes to reduce aflatoxin contamination in maize, and potentially in other economically important crop plants.

## Author Contributions

JC, KR, GP,DB, andRMconceived and designed the experiments. RM, CS, CC-W, and QW performed the experiments. BM, AF, MG, RM, CS, ML, and GP analyzed the data. RM, JC, KR, AF, and ML wrote the paper. All authors reviewed and approved the final manuscript.

## Conflict of Interest Statement

The authors declare that the research was conducted in the absence of any commercial or financial relationships that could be construed as a potential conflict of interest.

## Supplementary Material

FIGURE S1 Maize Interactome.

FIGURE S2 Co-expression network.

FIGURE S3 Transcriptional targets.
