# Supplementary figures and images for: The Pathogenesis-Related Maize Seed (PRms) Gene Plays a Role in Resistance to Aspergillus flavus Infection and Aflatoxin Contamination
Source: Front Plant Sci. 2017 Oct 17;8:1758. doi: 10.3389/fpls.2017.01758 (PMC5651032; doi:10.3389/fpls.2017.01758)

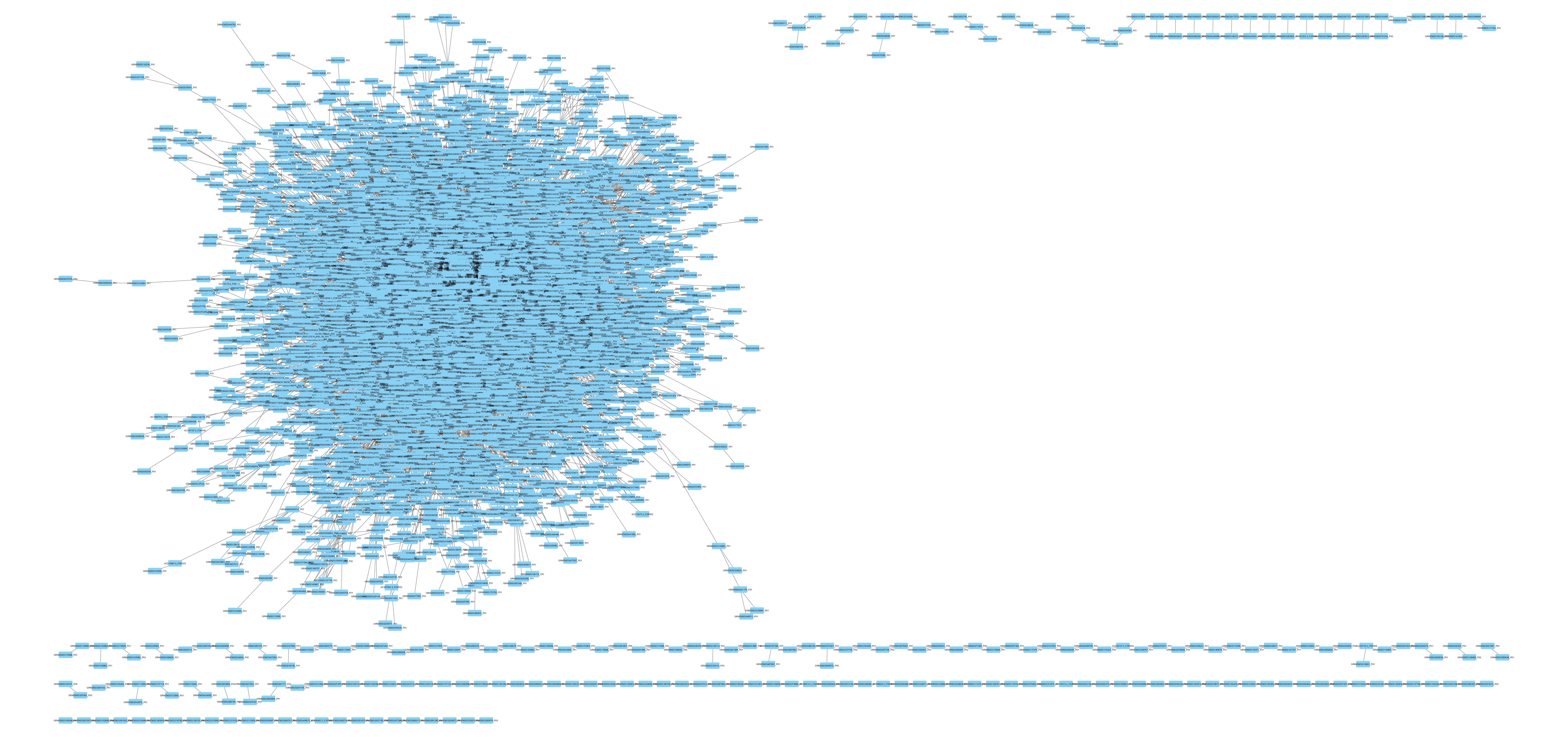

Supplement: FIGURE S1 — Maize Interactome. [file Image_1.PNG]

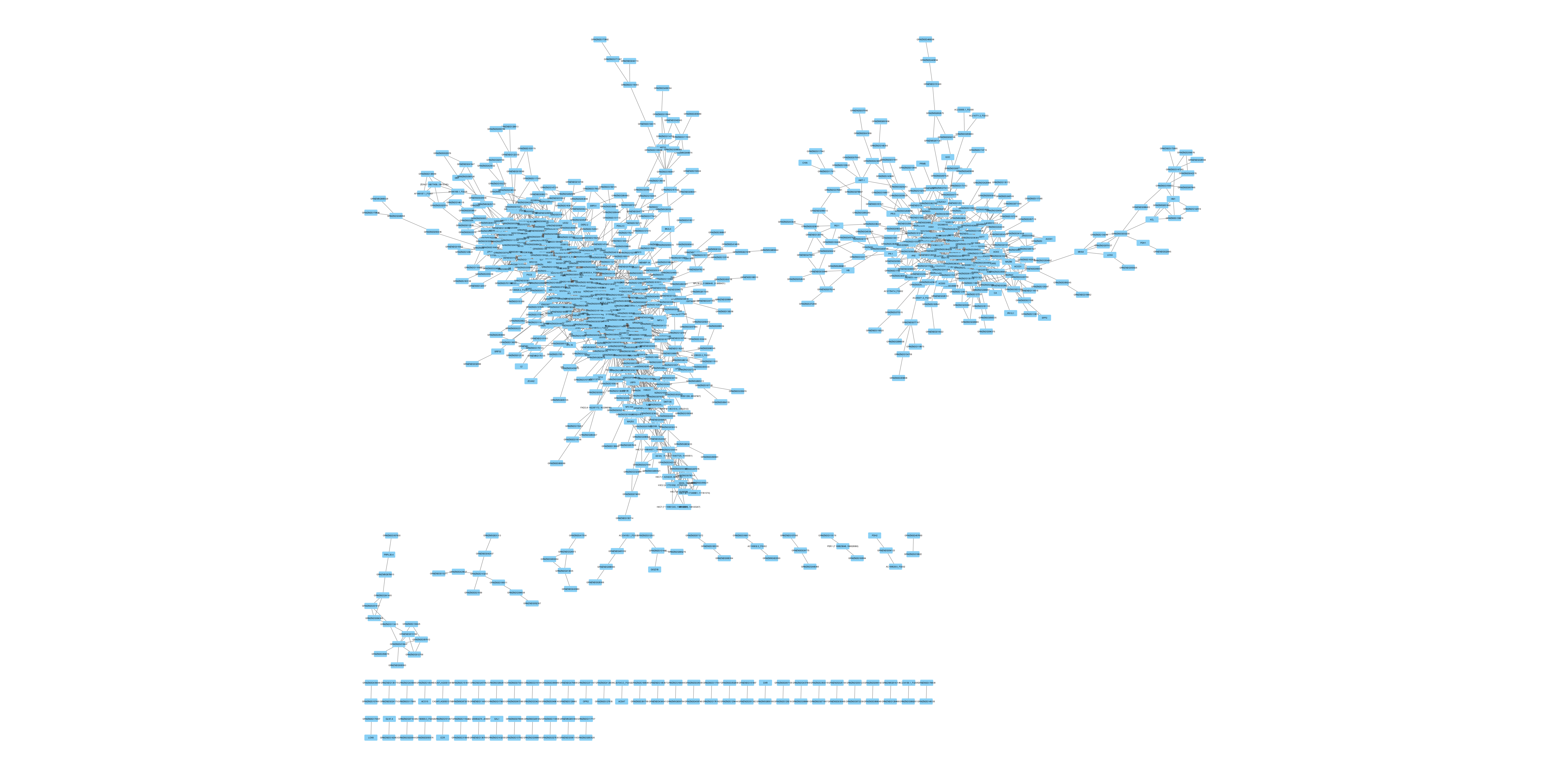

Supplement: FIGURE S2 — Co-expression network. [file Image_2.PNG]

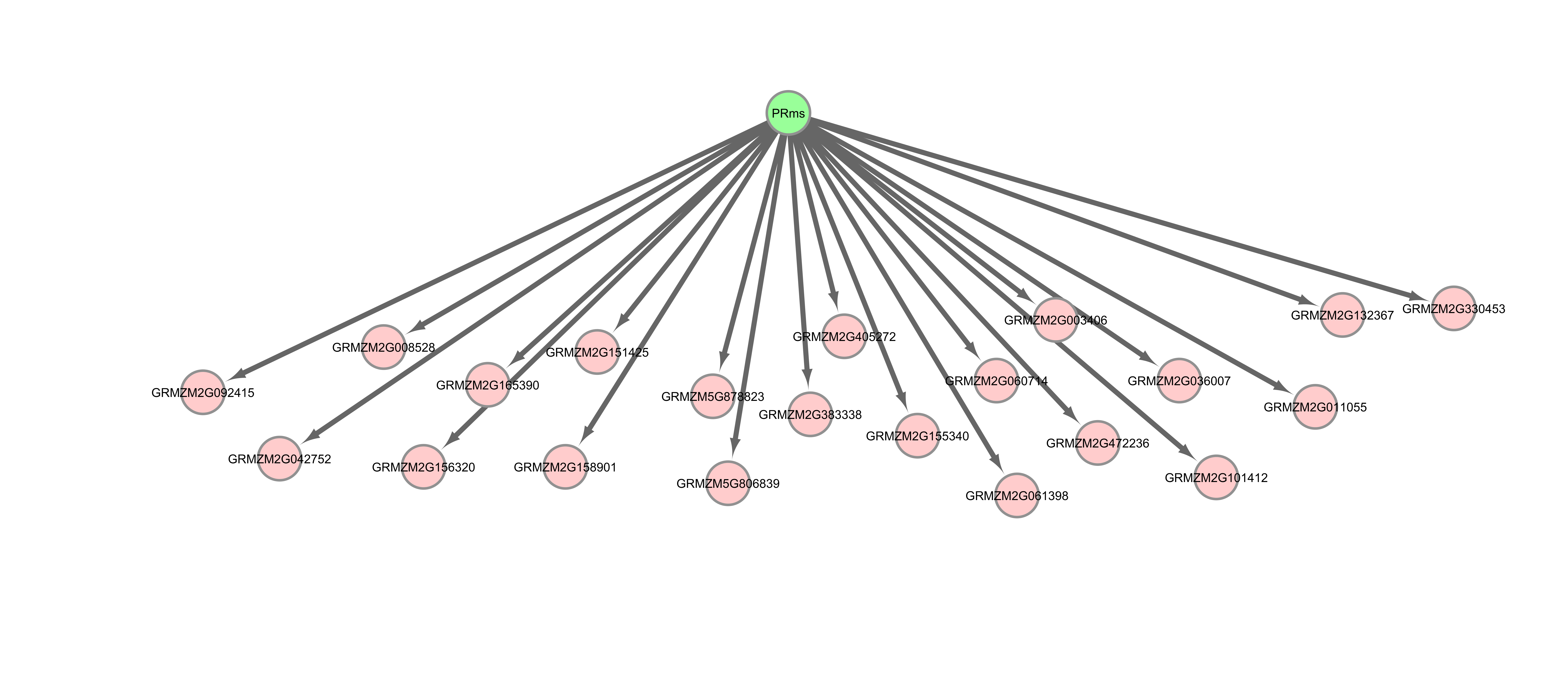

Supplement: FIGURE S3 — Transcriptional targets. [file Image_3.PNG]
