# Supplementary material for: The Pathogenesis-Related Maize Seed (PRms) Gene Plays a Role in Resistance to Aspergillus flavus Infection and Aflatoxin Contamination
Source: Front Plant Sci. 2017 Oct 17;8:1758. doi: 10.3389/fpls.2017.01758 (PMC5651032; doi:10.3389/fpls.2017.01758)
Supplement: Supplementary file 4 [file Table_1.pdf]

Table S1. List of primers used for cloning (vector construction) and qRT-PCR work.

Primer name	Forward (5'-3')	Reverse (5'-3')	Notes	Reference
Fragment 1 (F1)	AATATAATCTATAGTACTCGATCGTCCCGCGTCAAT	CTGCGAGCTTGTTGGGGTGTCGATCGGGTTCTT	Cloning	This study
Fragment 2 (F2)	CCAACAAGCTCGCAGTCT	GAGGTGAAGTTGAACGGGCTCGTAGTTGCAGAT	Cloning	This study
Fragment 3 (F3)	GTTCAACTTCACCTCAGGT	GTCTTTATCATCTGCAAGCTGAACGGCATGACTG	Cloning	This study
Fragment 4 (F4)	GCAGATGATAAAGACGCCA	CTCTAGACCCACTAGTATGGAGGCATCCAACAAGC	Cloning	This study
qPRms (AC205274.3_FG001)	TACAATGGAGGCATCCAACA	CTGTTTTGGGGAGTGAGGTA	qRT-PCR	Shu et al., 2015
qRib (GRMZM2G024838; house-keeping)	GGCTTGGCTTAAAGGAAGGT	TCAGTCCAACTTCCAGAATGG	qRT-PCR	Shu et al., 2015
qAnthocyanidin (GRMZM2G165390)	GCAAACAGGGTGGACGAG	ATGACGTAGTTGAAGTCGCC	qRT-PCR	This study
qF-Box (GRMZM2G008528)	GGAAACGGTACACTCTTACTCG	AGCAGACCATCGACCAAAG	qRT-PCR	This study
qInositol-Pase (GRMZM2G036007)	TCTTGGGATCACGCTGTTG	GCACCATTTGTCACCAGAAC	qRT-PCR	This study
Un1 (GRMZM2G042752)	GGAAACGGTACACTCTTACTCG	AGCAGACCATCGACCAAAG	qRT-PCR	This study
qLRR (GRMZM2G060714)	CCATGACAGTGTTCTCTTCCTG	CTAGTCACCAATTCACCCAGG	qRT-PCR	This study
qUn2 (GRMZM2G061398)	GCTCAGTCTACCAATGTTCCTC	ACCATCTTCCCTACCACTCC	qRT-PCR	This study
qUn3 (GRMZM2G092415)	GGTGTCTGTAGTCTGTCCATG	TCAGCATCAGCAAGTAGCAG	qRT-PCR	This study
qUn4 (GRMZM2G101412)	TCCCCACAATGTAATGCTGAG	ACTTCAATTCCCCTGGCTTG	qRT-PCR	This study
qHB-TF (GRMZM2G132367)	CACAGTACCCGTTCCCATTC	AGTTGAAGTTGAGGCTGGAG	qRT-PCR	This study
qAlpha-hydrolase (GRMZM2G151425)	GTTGAGTGCCTGATCTTTTCTG	ACACCATCCTCACAAACGAC	qRT-PCR	This study
qRabGAP (GRMZM2G156320)	TCAACGACTTTCAAGATCCCC	TCTAACATTATCCCCATGCCTG	qRT-PCR	This study
qtRNA-His-ase (GRMZM2G158901)	CAGCGAGTATGAGTACGTGAAG	CCTTTGAGAACCTGTGGAAGT	qRT-PCR	This study
qCholine transporter (GRMZM2G330453)	ATCCCTCTGGTATCCTCTGTC	CTTTTGGCGGGTTTGTGAC	qRT-PCR	This study
qUn6 (GRMZM2G383338)	TGGCAACACCAGTAGAAGATG	AAGGCCAGCATTGTGAAGA	qRT-PCR	This study
qEreb44 (GRMZM5G806839)	GGCTCAAGAGCTCCAAGAAA	GTGGACGAGCAGCAAGAG	qRT-PCR	This study
qMicrotubule (GRMZM5G878823)	CATTGCTGTGTTGCTGTTCTC	TCAATTTCGGAGGAGCCTTTAT	qRT-PCR	This study
qRab28 (GRMZM2G472236)	GGAGACGAGGACAAGGC	TCGTTCCTGTTGCGGTTC	qRT-PCR	This study
